# Management of Pruritus in Chronic Liver Disease

**DOI:** 10.1155/2015/295891

**Published:** 2015-03-10

**Authors:** Angeline Bhalerao, Gurdeep S. Mannu

**Affiliations:** ^1^General Practice, Ipswich, Suffolk IP4 5PD, UK; ^2^Oxford University Hospitals, Oxford OX3 9DU, UK

## Abstract

*Background.* There continues to be uncertainty on the ideal treatment of pruritus in chronic liver disease. The aim of this study was to gather the latest information on the evidence-based management of pruritus in chronic liver disease. *Methodology.* A literature search for pruritus in chronic liver disease was conducted using Pubmed and Embase database systems using the MeSH terms “pruritus,” “chronic liver disease,” “cholestatic liver disease,” and “treatment.” *Results.* The current understanding of the pathophysiology of pruritus is described in addition to detailing research into contemporary treatment options of the condition. These medical treatments range from bile salts, rifampicin, and opioid receptor antagonists to antihistamines. *Conclusion.* The burden of pruritus in liver disease patients persists and, although it is a common symptom, it can be difficult to manage. In recent years there has been greater study into the etiology and treatment of the condition. Nonetheless, pruritus remains poorly understood and many patients continue to suffer, reiterating the need for further research to improve our understanding of the etiology and treatment for the condition.

## 1. Introduction

Pruritus or itch is a common symptom seen in a number of illnesses. It is an unpleasant sensation of irritation of the skin. Pruritus can further be classified as localised or generalised depending on the affected area and acute or chronic depending on the duration of the symptom. Chronic pruritus is defined as presence of pruritus for more than 6 weeks. Pruritus associated with liver disease has been well described as early as the 2nd century BC when the Greek physician Aretaeus the Cappadocian observed an association between pruritus and jaundice [1]. Pruritus is a common clinical feature seen in most liver diseases but particularly frequently in cholestatic liver disease. Cholestatic liver disease can be further classified into intra- and extrahepatic disease. Chronic pruritus is more frequently seen in intrahepatic cholestatic diseases such as primary biliary cirrhosis (PBC), intrahepatic cholestasis of pregnancy, chronic hepatitis B and C, familial intrahepatic cholestasis, and Alagille syndrome. However, pruritus is also seen in extrahepatic cholestatic liver diseases such as primary sclerosing cholangitis (PSC) and cancer of the head of pancreas [2].

Pruritus contributes a large symptomatic burden to those suffering from liver diseases. A recent survey reported that pruritus occurs in 69% of PBC sufferers and, for 75% of these patients, pruritus was present before the diagnosis of PBC, possibly suggesting pruritus as a diagnostic criterion. Pruritus in PBC can be very debilitating as approximately 65% of PBC sufferers report itching to occur especially at night time, thus affecting sleep. In some PBC patients, pruritus is worse after meals and premenstrually [3]. Cholestasis, pruritus, and jaundice are the main clinical features of progressive familial intrahepatic cholestasis [4]. Furthermore, 15–31% of hepatitis C sufferers complain of chronic pruritus. In all of these cases, pruritus in chronic liver disease tends to be generalised, chronic, intermittent, and of varying severity. It adversely affects patient's quality of life by frequently disrupting sleep [5], their daily activities, and personal relationships. It can also lead to depression and even suicidal intent in extreme cases [6]. Due to the subjective nature of pruritus there is an added difficulty in determining its severity and in treating it. Due to incompletely understood etiology and various different treatments available for pruritus, there remains ambiguity regarding the ideal approach to the treatment of this condition. In light of this, this review aimed to collate all published literature on the pathophysiology and management of pruritus in chronic liver disease in order to address this issue.

## 2. Methodology

A literature search for pruritus in chronic liver disease was conducted using Pubmed and Embase database systems using the MeSH terms “pruritus,” “chronic liver disease,” “cholestatic liver disease,” and “treatment.” This is summarised in supplementary figure  1 in Supplementary Material available online at http://dx.doi.org/10.1155/2015/295891.

### 2.1. Eligibility Criteria

All prospective and retrospective studies that recruited patients of any age and identified pruritus through clinical assessment were selected. Relevant studies needed to have a longitudinal follow-up of at least 24 hours duration and to report on pathophysiology, treatment, or outcomes. Papers were restricted to patients with chronic liver disease alone.

### 2.2. Information Sources and Search Strategy

In January 2014 a systematic search utilising PubMed/Medline and OVID search engines was conducted. The initial search was undertaken using MESH search for “pruritus” and “liver disease” and key phrases as listed in supplementary Figure  1. To capture the most recent literature in the field and to ensure that our analysis was based on contemporary datasets, the time period of literature search was limited to the past 20 years (January 1994–January 2014). The results of papers focusing on management were limited to papers focusing on human subjects and in the English language.

### 2.3. Study Selection

The abstracts were screened and relevant articles meeting the above criteria were selected. Searches were conducted by the authors who independently checked titles and abstracts against the eligibility criteria and subsequently obtained full-text versions of all potentially relevant papers, which were then further considered for final inclusion.

## 3. Results

### 3.1. Pathophysiology

The exact pathogenesis of pruritus in chronic liver disease is unknown; however, several hypotheses have been suggested. Pruritus induced by certain substances known as pruritogens is one of the implicated theories. Several pruritogens have been identified over the years. The “bile salts theory” proposes bile salts as the pruritogen. Cholestatic liver disease increases levels of bile salt which accumulate under the skin causing itch. This theory is further supported by studies showing that ingestion of bile salts in cholestatic patients worsens pruritus [6, 7] and intradermal injection of bile salts causes pruritus in healthy persons [8, 9]. Additionally, when bile is removed through nasobiliary drainage or partial external biliary diversion in a cholestatic patient, pruritus is significantly reduced [10, 11]. However, there still exists no established correlation between the bile salt concentration and severity of pruritus [12, 13]. Furthermore, not all cholestatic patients with elevated levels of bile salts experience pruritus [14] and, additionally, pruritus also occurs in patients with normal levels of bile salts [12].

Histamine is also one of the strong contenders as a pruritogen in cholestatic pruritus. Raised histamine levels are found in cholestatic pruritus sufferers [15]; however, again there is no correlation between histamine concentrations and severity of pruritus [16] and antihistamines are often ineffective in treating pruritus in this setting [17]. Opioids, serotonin, and female sex hormones have all been implicated in the etiology of pruritus. Increased levels of endogenous opioids are reported in chronic liver disease [18, 19] and treatment with an opioid antagonist is shown to reduce pruritus [20–22]. Serotonin is believed to induce pruritus by altering itch perception [17] and so serotonin reuptake inhibitors such as sertraline have reported to be effective in managing pruritus [23].

Female hormonal influence on cholestatic pruritus is seen in different liver diseases. The intrahepatic cholestatic pruritus of pregnancy is self-limiting and often resolves after pregnancy. In addition, symptoms in preexisting primary biliary cirrhosis and primary sclerosing cholangitis female sufferers can sometimes worsen during pregnancy when there are raised female sex hormones [7]. Generally, increased itch sensation is apparent during pregnancy and in women taking hormone replacement therapy [8].

Recent research on cholestatic pruritus have identified another pruritogen called lysophosphatidic acid (LPA). Lysophosphatidic acid is a phospholipid which affects a range of cellular functions. Autotaxin (ATX) is an enzyme which cleaves lysophospholipase to form LPA. Both LPA and autotaxin levels are raised in patients with cholestatic pruritus. Additionally, studies on mice reveal that intradermal injections of LPA produce a dose-dependent induction of pruritus [16, 24]. Pregnane X receptor (PXR) which is a nuclear steroid receptor is believed to have a vital role in ATX synthesis; however, the mechanism still remains unclear. In vitro studies have shown that the PXR agonist rifampicin reduces ATX synthesis and hence reduces pruritus [9].

In terms of the transduction of pruritus sensation, there are two main theories. The first is the intensity theory which proposes that the same neuronal pathways carry both the itch and pain stimuli. As a result, a weaker stimulus gives an itch perception and an increased stimulus gives the perception of pain. The second is the specificity theory, which suggests that a different group of nerves carries the itch and pain perception separately and factors such as genetics, diet, and environment may be responsible for varying susceptibility for pruritus between individuals.

### 3.2. Management of Pruritus

There has been a plethora of work investigating possible treatment options for pruritus in the setting of chronic liver disease. These medical treatments range from bile salts, rifampicin, andopioid receptor antagonists to antihistamines. Additionally nonpharmacological management such as skin moisturisers, avoidance of skin irritants, and avoiding hot environments can also prove to be very beneficial in reducing pruritus.

Bile salt resins such as cholestyramine are usually the first line treatment for pruritus in cholestatic disease. Several studies have shown the efficacy of bile salt resins in symptom control of pruritus [10, 11]. Cholestyramine is an effective medication with minimal side effects, which include gastrointestinal upset, unpleasant taste, and rarely fat malabsorption. Ursodeoxycholic acid (UDCA) is one of the bile acids which has been shown to improve jaundice, improve ascites, and improve liver function in primary biliary cirrhosis [12], however, has little benefit on pruritus [13]. It is, however, highly effective in intrahepatic cholestasis of pregnancy (ICP) [14] and hence UDCA is currently only indicated in the treatment of ICP in light of a recent randomised control trial which showed that UDCA improves pruritus and is safe to use during pregnancy [18]. More recent research has explored farnesoid X nuclear receptors in maintaining homeostasis in bile acid synthesis and farsenoid X receptor agonists may prove to be an upcoming treatment option for PBC [19].

Rifampicin is another effective treatment option for cholestatic pruritus, especially in pruritus refractory to therapy and in malignant cholestasis [20, 21]. A recent meta-analysis of randomized controlled trials highlighted the safety of rifampicin in the treatment of cholestatic pruritus [22]. However, regular blood test monitoring is still needed for patients on rifampicin treatment owing to the risk of hepatotoxicity [16]. *μ*-Opioid receptor antagonists such as naloxone or naltrexone are also shown to be effective in the management of cholestatic pruritus [15, 24, 25]. However, opiate withdrawal reaction is one of the common side effects and hence this treatment option should be avoided in patients with drug addiction issues [25]. It should also be avoided in patients with acute hepatitis and liver failure. Finally, in a placebo-controlled trial, selective serotonin reuptake inhibitor sertraline was shown to be more effective than placebo group in controlling pruritus [23].

Contrary to established doctrine, a recent review has shown that topical antihistamines are not very effective in the treatment of pruritus [26]. There are still new emerging therapeutic options for pruritus treatment for patients who remain refractory to the abovementioned treatments. Although further evidence is needed to further test their efficacy. Albumin dialysis using molecular adsorbent recirculating system is one of them. A multicentric analysis concluded that the dialysis was significantly effective in pruritus management [27]. Similarly plasmapheresis is suggested as a treatment option for primary biliary cirrhosis in pregnant woman [28].

There are several other potentially useful agents in the management of chronic liver disease-associated pruritus but to date have only been confined to isolated case reports and small-scale series and so cannot be recommended. However, these are discussed here for completeness and include thalidomide, ondansetron, phenobarbital, and stanozolol. Thalidomide is an example of a primary antipruritic agent which has shown promise in primary biliary sclerosis. Its side effects can include significant drowsiness, suggesting a central depressant mechanism underlying it action [1, 2]. Ondansetron is a serotonin 5-HT3 receptor subtype antagonist that is effective in the management of nausea and vomiting. Although it is usually tolerated well with few side effects, there is only anecdotal evidence to support its use in pruritis from chronic liver disease and studies have provided mixed results [3–5]. Similarly, phenobarbital or phenobarbitone is a long-acting barbiturate and has also been investigated in reducing pruritus in chronic liver disease; however, it also does not appear to have a clear beneficial effect [6, 17]. Stanozolol is a synthetic anabolic steroid derived from dihydrotestosterone. Although it relieves pruritus, it also worsens cholestasis and so cannot be recommended [23].

## 4. Discussion

The impact of pruritus on the quality of life of patients suffering from chronic liver failure is often underestimated by physicians. Although the severity of pruritus is variable between patients, it can have significant implications on a patient's mental health and psychological well-being. The paucity of clinical literature addressing pruritus in liver patients demonstrates the lack of focused research on the topic and in turn highlights the difficulty faced by the physician when confronted with treatment-resistant patients with pruritus.

The underlying pathophysiology is unclear and is likely to be a result of a number of interrelated complex pathways with multifactorial etiologies [29]. The European Association for the Study of the Liver (EASL) has established guidelines for the initial clinical assessment, investigation, and management of pruritus in cholestatic liver diseases [30]. The approach to management should be in a step-wise fashion starting with the simple agents listed above and then escalating to more experimental treatments in resistant cases. An appropriate approach would be to start with UDCA and then cholestyramine followed by rifampicin and naltrexone and if symptoms persist this may be followed by therapies such as sertraline [31]. Experimental therapies such as UVA/B light therapy or other experimental drug therapies can be reserved for cases resistant to conventional therapy [32].

Hence it is clear that, due to poorly understood pathophysiology, there is no one single ideal treatment for all chronic liver disease patients suffering from pruritus. Although there are several treatment options available, achieving optimum symptom control may require a trial and error process to find the best regime for each patient. Nonetheless, despite available treatments a small number of sufferers may not respond to any therapy and this group may require liver transplant, even in the absence of liver failure, to treat their symptoms [30, 33, 34].

This review was restricted to published literature in the English language and confined to the eligibility criteria described in the methods section. Due to the heterogeneity of the outcomes measured in the literature and the wide remit of this review a quantitative analysis was not feasible. Nonetheless the general conclusions from the current evidence base have been presented. The future of experimental research in this field will focus on novel agents in the treatment of pruritus; however, basic research into understanding the underlying etiology and signaling of pruritus is paramount for pharmacological progress in this field. Nonetheless there is also a clear need for focused work in phase III and IV studies comparing the clinical effectiveness of established agents and combinations thereof in different etiologies of liver disease and different patient subgroups in order to strengthen the evidence base upon which clinical guidelines can be set.

## 5. Conclusion

The burden of pruritus in liver disease patients persists and although it is a common symptom, it can be difficult to manage. Despite there being a large body of research into the etiology and treatment of the condition, pruritus remains poorly understood and many patients continue to suffer. What is known has been presented in this review but the field requires continued basic science research to help broaden our knowledge of the etiology of pruritus and more clinical research on treatment options to help improve the quality of life of chronic liver disease patients.

## Supplementary Material

Supplementary Figure 1: In January 2014 a systematic search for pruritus in chronic liver disease was conducted using PubMed/Medline and Embase database systems using the MeSH terms “pruritus,” “chronic liver disease,” “cholestatic liver disease,” and “treatment.” The process of paper selection is illustrated in this figure. All prospective and retrospective studies that recruited patients of any age and that identified pruritus through clinical assessment were selected. Relevant studies needed to have a longitudinal follow-up of at least 24 hours duration and to report on pathophysiology, treatment, or outcomes. Papers were restricted to patients with chronic liver disease alone. To capture the most recent literature in the field and to ensure that our analysis was based on contemporary datasets, the time period of literature search was limited to the past 20 years (January 1994–January 2014). The results of papers focusing on management were limited to papers focusing on human subjects and in the Eng-lish language.
