# Supplementary material for: Management of Pruritus in Chronic Liver Disease
Source: Dermatol Res Pract. 2015 Mar 10;2015:295891. doi: 10.1155/2015/295891 (PMC4377431; doi:10.1155/2015/295891)
Supplement: Supplementary file 1 — Supplementary Figure 1: In January 2014 a systematic search for pruritus in chronic liver disease was conducted using PubMed/Medline and Embase database systems using the MeSH terms “pruritus,” “chronic liver disease,” “cholestatic liver disease,” and “treatment.” The process of paper selection is illustrated in this figure. All prospective and retrospective studies that recruited patients of any age and that identified pruritus through clinical assessment were selected. Relevant studies needed to have a longitudinal follow-up of at least 24 hours duration and to report on pathophysiology, treatment, or outcomes. Papers were restricted to patients with chronic liver disease alone. To capture the most recent literature in the field and to ensure that our analysis was based on contemporary datasets, the time period of literature search was limited to the past 20 years (January 1994–January 2014). The results of papers focusing on management were limited to papers focusing on human subjects and in the Eng-lish language. [file 295891.f1.docx]

Supplementary Figure 1: Data selection flowchart: Demonstrating the pathway of paper selection.

Potentially relevant studies identified and screened for retrieval using key words ’ (n=2,423)

Studies excluded, with reasons Humans, English, published in the last 20 years (n= 33248)

Potentially appropriate studies to be included in the Systematic review (n=51) included in qualitative synthesis

Studies not relating to pruritus or liver disease on screening abstracts (n= 2372)

Potentially relevant studies identified and screened for retrieval using key word "pruritus" and ‘chronic liver disease’ [Mesh] using PUBMED and OVID (n= 35, 671)
